# Synthesis, Characterization, and Antifungal Studies of Cr(III) Complex of Norfloxacin and Bipiridyl Ligand

**DOI:** 10.1155/2014/457478

**Published:** 2014-09-03

**Authors:** Anamika Debnath, Firasat Hussain, Dhanraj T. Masram

**Affiliations:** Department of Chemistry, University of Delhi, Delhi 110007, India

## Abstract

A novel slightly distorted octahedral complex of Cr(III) of norfloxacin (Nor) with the formula [Cr^III^(Nor)(Bipy)Cl_2_]Cl*·*2CH_3_OH has been synthesized hydrothermally in the presence of a *N*-containing heterocyclic compound 2,2′-bipyridyl (Bipy). The complex was characterized with FT-IR, elemental analysis, UV-visible spectroscopy, and X-ray crystallography. Spectral studies suggest that the Nor acts as a deprotonated bidentate ligand. Thermal studies were also carried out. The synthesised complex was screened against four fungi *Pythium aphanidermatum* (PA), *Sclerotinia rolfsii* (SR), *Rhizoctonia solani* (RS), and *Rhizoctonia bataticola* (RB).

## 1. Introduction

**Figure 1:**
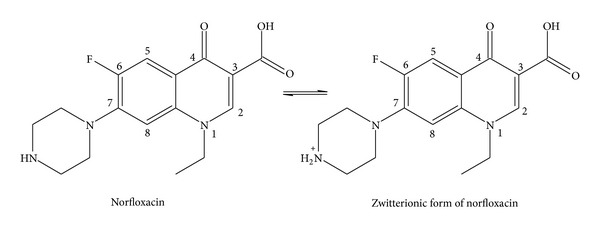
Molecular structure of Nor and its zwitterion.

Quinolones are the broad spectrum synthetic antibiotics containing 4-oxo-3-carboxylic-1,4-dihydroquinoline skeleton, with bactericidal effect, good oral absorption, excellent bioavailability, and good penetration into tissues [1, 2]. Nalidixic acid is the first member of quinolones [3]. Due to the narrow spectrum of nalidixic acid several modifications were made on the basis of structure activity relationship (SARS) to enhance the bactericidal spectrum and improve the pharmacokinetics properties. It has been found that the introduction of fluorine atom at position 6 and a piperazine ring at position 7 without the presence of N at position 8 enhances the biological activity spectrum. The quinolones with these modifications are grouped together as fluoroquinolones. Norfloxacin (Nor) (Figure 1) is the first member of the fluoroquinolones. This quinolone usually acts as bidentate ligand due to the presence of pyridone oxygen at position-4 and one of the carboxylate oxygens at position-3 and it exists in a zwitterionic form. Treatment with this drug leads to uncoiling of double stranded DNA and causes immediate microbial cell death [4, 5].

In the last few years, the synergetic effect of transition metal ions with quinolones has been the subject of active research in bioinorganic chemistry. It has been observed that metal complexes with appropriate ligands are biologically more significant and specific than the metal ions and the ligand itself [6–8]. Utilization of metal ions in treatment of various diseases has been thoroughly studied and a large number of quinolone metal complexes with diverse metal ions have been reported [9]. The majority of the metal-quinolone complexes are neutral with metal ions such as magnesium(II) [10], calcium(II) [11], boron(III) [12], vanadium(IV) [13], manganese(II) [14], iron(III) [15], cobalt(II) [16], nickel(II) [17], copper(II) [18], zinc(II) [19], silver(I) [20], cadmium(II) [21], cerium(III) [22], and lead(II) [23], while for magnesium(II) [24], iron(III) [25], copper(II) [26], zinc(II) [26], platinum(II) [27], and bismuth(III) [28], the complexes are either binary metal-quinolone complexes or ternary complexes with one or more O—(e.g., H_2_O, MeOH, and DMSO) or N-donor ligands (e.g., pyridine, 2,2′-bipyridine) as coligands. There are some Ru(III) complexes of quinolones with potential antitumor activity that have also been reported [29]. Anticancer potency of Ru(III) complexes containing antibacterial quinolones was reported [30].

In order to investigate the coordination behaviour of Nor we have synthesised a novel mixed ligand complex of Cr(III) metal ion in presence of* N*-containing heterocyclic ligand Bipy hydrothermally by gradual heating and cooling.

During literature survey it has been found that all of the reported synthesised metalloquinolones are screened against animal pathogenic microbes (fungus and bacteria). In this present work we have screened our synthesised complex against four phytopathogenic fungi for suppression of various plant diseases to develop the agriculture science by protecting crops and vegetables for the production of a plentiful supply of high-quality and affordable food.

## 2. Experimental

### 2.1. Materials

Nor was purchased from Sigma Aldrich. Bipy and the metal salt CrCl_3_ *·*6H_2_O were obtained from Merck. All the chemicals used for this work were of analytical grade.

### 2.2. Synthesis of the Complex

An equimolar mixture of CrCl_3_ *·*6H_2_O and Bipy in 15 mL of 1 : 1 solvent mixture of methanol and acetone was stirred for 10 minutes on a magnetic stirrer at room temperature. Then 5 mL equimolar solution of Nor in the same solvent mixture was added drop by drop under stirring condition. Further, the resulting mixture was heated in a hydrothermal vessel in programmed temperature oven at 100°C for 24 hrs. Then it was gradually cooled to room temperature after 72 hrs, leading to a bright green needle shaped crystal.

Calc. for C_28_H_35_Cl_3_CrFN_5_O_5_: C, 48.11; H, 5.05; N, 10.02%. Found: C, 48.40; H, 4.91; N, 10.61%.

### 2.3. Physical Measurements

Fourier transform infrared (FT-IR) spectra were recorded on a spectrometer Perkin Elmer Spectrum BX II in the range of 400–4000 cm^−1^ by preparing sample pellets with KBr. Electronic spectra were recorded in solid state on an instrument Shimadzu UV-3101PC spectrometer. C, H, and N elemental analysis was performed on an Elementer vario ELIII instrument. Thermogravimetric analysis (TGA) measurements were carried out in an oxygen atmosphere from ambient temperature to 900°C using Perkin Elmer Diamond. Single-crystal X-ray diffraction (XRD) was collected in a Bruker D8 diffractometer, using Cu Kα radiation.

### 2.4. Microbiological Studies

The* in vitro* antifungal activities of the ligand and the synthesised complex have been evaluated against four pathogenic fungi,* Pythium aphanidermatum* (PA),* Sclerotinia rolfsii* (SR),* Rhizoctonia solani* (RS), and* Rhizoctonia bataticola* (RB), by the agar plate technique. The compounds are directly mixed with the medium in 0, 0.0125, 0.025, 0.05, and 0.1 mg/mL (in 0.1% of Dimethyl sulfoxide) concentrations. Controls were also run and three replicates were used in each case. The antimicrobial activity is estimated on the basis of the size of the inhibition zone around the dishes after four days and the percentage inhibition was calculated by the following equation:

(1) $$\begin{array}{c} \%\text{inhibition}=\left(C-\frac{T}{C}\right)*100, \end{array}$$

where *C* and *T* are the diameters of the fungal colony in the control and the test plates, respectively [31].

## 3. Results and Discussion

### 3.1. Infrared Spectroscopy

FT-IR band assignments were done by comparing the spectra of the synthesised complex with those of the free ligand, Nor. FT-IR spectra of free ligand in KBr disk show that the peak at 1617 cm^−1^, assigned for pyridine stretch *ν*_(C=O)py_, was slightly shifted to the 1630 cm^−1^ in the complex (Supplementary Figure S1 in Supplementary Materials available online at http://dx.doi.org/10.1155/2014/457478). In case of free ligand, a characteristic absorption band at ~1733 cm^−1^which is assigned for carboxylic stretch *ν*_(C=O)carb_, was replaced in the complex by two characteristic bands at 1587 cm^−1^ and at 1381 cm^−1^, assigned as asymmetric *ν*(O–C–O)a and symmetric *ν*(O–C–O)s stretching vibrations, respectively [6]. This indicates the involvement of the pyridone oxygen and carboxylate oxygen in the coordination with Cr(III) ion. The difference Δ*ν* = *ν*(O–C–O)a − *ν*(O–C–O)s is the important criteria for the determination of coordination mode of the ligand [32]. Δ*ν* of the complex was found to be 206 cm^−1^ that indicates the monodentate interaction of the carboxylate group with metal ion. The FT-IR data of the complex shows a very strong and broad band at 3368 cm^−1^ and medium to weak bands at 2841 and 2487 cm^−1^. These three bands confirm the vibration of quaternized nitrogen of the piperazinyl group indicating that the zwitterionic form of free Nor involves during complex formation with the Cr(III) ion.

### 3.2. Electronic Spectra

Electronic spectra of the synthesized Cr(III) metal complex and the free ligand Nor were recorded in the range of 200–900 nm in solid state. Two bands have been found at 285 nm and 335 nm in case of free ligand. These two bands were assigned to *π*-π* and *n*-π* transitions, respectively. These two transitions were observed due to the presence of aromatic ring containing pyridone oxygen and carboxylate oxygen. Pattern of the electronic spectra of Cr(III) complex is similar to that of the free ligand, indicating that the ligand has not changed its structure during complexation; differences due to Bipy are not easily distinguished [33–36]. The band at 285 nm in the spectra of the complex is shifted hypochromically compared to the free ligand and the band at 335 nm slightly shifted to higher wave length region (Supplementary Figure S2), suggesting that both pyridone oxygen and carboxylate oxygen participate in the complex formation. A broad band was observed in the visible region which is centred at 590 nm due to d-d transition.

### 3.3. Thermal Analysis

**Figure 2:**
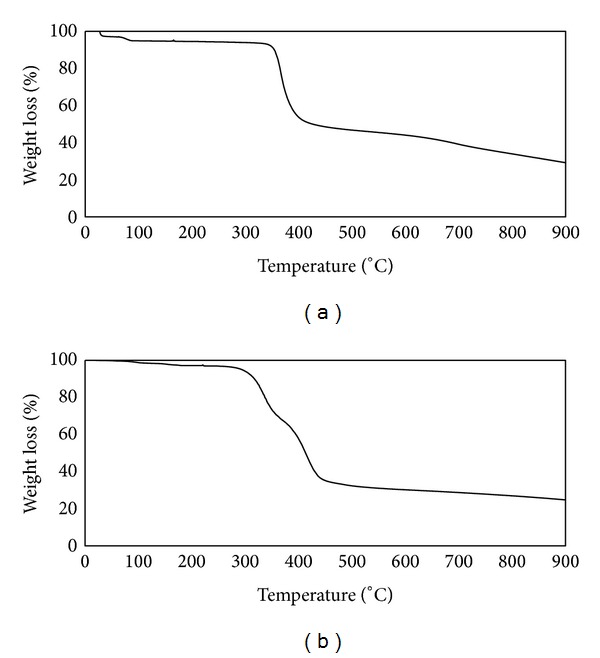
TGA pattern of (a) Nor and (b) [Cr(Nor)(Bipy)Cl_2_]Cl*·*2CH_3_OH.

Thermal analysis of metal complex and free ligand was also studied starting from ambient temperature to 900°C with controlled heating rate of 10°C min^−1^ under oxygen atmosphere. The temperature ranges, percentage weight loss, eliminated moiety of every decomposition, and melting point are listed in Table 1. The free ligand and the metal complex were found to have three and four stages of weight loss, respectively (Figure 2). In case of free ligand 9% of weight loss was observed in the temperature range of 25–273°C. Second step of weight loss started at 273°C and ended at 579°C with 70.63% of weight loss and third decomposition occurs between 579°C and 727°C with a weight loss of 20.25%. In case of metal complex first weight loss was observed in the temperature range 31–101°C with 2.8% of weight loss. After that second step starts at 101°C and ends at 304°C with a mass loss of 5%. The third step was observed in the temperature range 304–508°C with weight loss of about 47.51% and forth step of weight loss occurs in the temperature between 508°C and 902°C with 17.47% of weight loss.

It has been also observed that the melting point of the synthesized complex (372°C) is more than that of parent quinolone (221°C). From the above thermal study, it is obvious that the synthesized complex is more thermally stable than its parent ligand Nor.

### 3.4. Crystal Structure of the Complex [Cr(Nor)(Bipy)Cl_2_]Cl*·*2CH_3_OH

A single crystal suitable for X-ray diffraction for compound [Cr(Nor)(Bipy)Cl_2_]Cl*·*2CH_3_OH was mounted on a capillary tube for indexing and intensity data collection at 183(2) K on an Oxford Xcalibur CCDC single-crystal diffractometer (MoKα radiation, *λ* = 0.71073 Å); see Table 2. Routine Lorentz and polarization corrections were applied, and an absorption correction was performed using the ABSCALE 3 program [37]. Direct methods were used to locate the heavy metal atoms (SHELXS-97). The remaining atoms were located from successive Fourier maps (SHELXL-97) [38].

**Figure 3:**
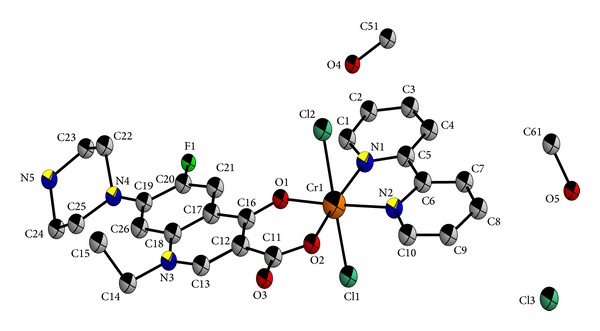
ORTEP diagram (50% probability factor for thermal ellipsoid) of the molecule [Cr(Nor)(Bipy)Cl_2_]Cl*·*2CH_3_OH; color code: carbon: grey; nitrogen: blue; oxygen: red; fluorine: green; chlorine: dark green; chromium: orange (only hydrogen atoms were not shown for clarity).

In the synthesised complex [Cr(Nor)(Bipy)Cl_2_]Cl*·*2CH_3_OH, Nor behaves as a bidentate ligand and is coordinated to Cr (III) through pyridone oxygen and one of the carboxylate oxygen atoms. The crystal structure is shown in Figure 3 and selected bond distances and bond angels are listed in Table 3. It has been found that in the complex Cr(III) ion is hexacoordinated surrounded by one pyridone oxygen, one of the carboxylate oxygen atoms, two ring N atoms of Bipy, and two chlorine atoms giving a distorted octahedral geometry.

Further details on the crystal structure data may be obtained from Cambridge data base, Oxford, on quoting the depository number* CCDC-953445*. Alerts shown in checkcif are due to disorder of solvent molecule.

### 3.5. Biological Activity

**Figure 4:**
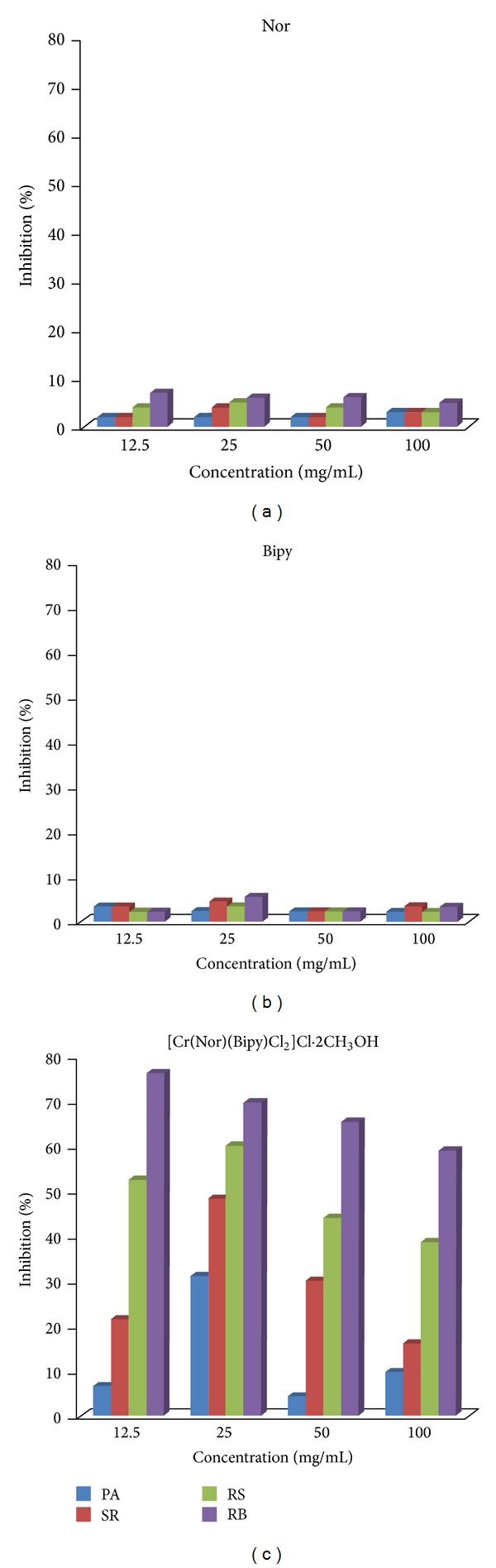
Percentage inhibition of (a) Nor, (b) bipy, and (c) [Cr(Nor)(Bipy)Cl_2_]Cl*·*2CH_3_OH against PA, SR, RS, and RB.

Antifungal activity of the free ligand and the complex against selected four pathogenic fungi* Pythium aphanidermatum* (PA),* Sclerotinia rolfsii* (SR),* Rhizoctonia solani* (RS), and* Rhizoctonia bataticola* (RB) was carried out. These four are soil born phytopathogenic fungi and are responsible for several plant diseases like damping-off of seedlings, stem canker, crown blight, root, crown, bulb, tuber, and fruit rots. In the future, our compound may become commercially available in order to improve the crop diseases control. The results of the antifungal tests are illustrated graphically in Figure 4. It has been observed that the antifungal activity of the newly synthesized complex increases with an increase in concentration, but remains the same for the parent ligand.

## 4. Conclusion

The synthesis and the characterization of neutral mononuclear mixed ligand metal complex of Nor and Bipy with Cr(III) metal ion have been realized with physicochemical and spectroscopic method. Nor coordinated with Cr(III) in a monodentate way and possesses distorted octahedral geometry. The complex shows more activity as compared with the standard ligand indicating that metal complexation enhances the activity of the parent ligand; this may be explained by chelation theory [37] according to which chelation reduces the polarity of the ligand and the central metal atom because of the delocalization of *π* electrons over the whole chelate ring increases, which favours permeation of the complexes through the lipid layer of the cell membrane.

## Supplementary Material

S1: FT-IR spectra of (a) Nor and (b) [Cr(Nor)(Bipy)Cl2]Cl.2CH3OH
S2: Electronic spectra of (a) Nor and (b) [Cr(Nor)(Bipy)Cl2]Cl.2CH3OH

## Figures and Tables

**Table 1:** Thermogravimetric data of Nor and [Cr(Nor)(Bipy)Cl_2_]Cl*·*2CH_3_OH.

Entry	Step	Temp. range (°C)	Weight loss (%)	M. P (°C)
nor	First	25–273	9.0	221
	Second	273–579	70.63	
	Third	579–727	20.25	

[Cr(Nor)(Bipy)Cl_2_]Cl*·*2CH_3_OH	First	31–101	2.82	372
	second	101–304	3.78	
	Third	304–508	47.31	
	Fourth	508–902	17.47	

**Table 2:** Single crystal X-ray crystallographic data for compound [Cr(Nor)(Bipy)Cl_2_]Cl*·*2CH_3_OH.

Empirical formula	C_28_H_34_Cl_3_CrFN_5_O_5_	*Z*	2
Formula weight	697.95	*ρ*_calcd_ [g cm^−3^]	1.532
Crystal system	Triclinic	**μ** [mm^−1^]	0.696
Space group Crystal color Temp.	P-1 Green 293 (2)	Reflection collected	15448
*a* [Å]	10.6127 (6)	Unique (Rint)	4711
*b* [Å]	12.0733 (7)	Observed [*I* > 2*σ*(*I*)]	
*c* [Å]	13.5624 (8)	Parameters	400
*α* [°]	68.548 (5)	Gof	1.035
*β* [°]	69.524 (5)	*R*[*I*>2*σ*(*I*)]^[a]^	0.0519
*γ* [°]	85.285 (4)	*R*_*w*_ (all data)^[b]^	0.1352
*V* [Å^3^]	1513.10 (15)		

^[a]^ *R* = ∑||*F*o| − |*F*c||/∑|*F*o|. ^[b]^ *R*_*w*_ = [∑*w*(*F*o^2^−*F*c^2^)^2^/∑*w*(*F*o^2^)^2^]^1/2^.

**Table 3:** Bond lengths (Å) and angles (°) of [Cr(Nor)(Bipy)Cl_2_]Cl*·*2CH_3_OH around Cr(III).

Angles	(Å)	Bonds	(°)
Cl_1_–Cr_1_–Cl_2_	177.51(4)	Cr_1_–N_1_	2.067(3)
Cl_1_–Cr_1_–O_1_	90.55(8)	Cr_2_–N_2_	2.052(3)
Cl_2_–Cr_1_–O_2_	88.92(8)	Cr_1_–O_1_	1.944(2)
Cl_1_–Cr_1_–N_1_	90.03(8)	Cr_1_–O_2_	1.934(2)
N_2_–Cr_1_–Cl_2_	90.58(8)	Cr_1_–Cl_1_	2.3288(10)
N_1_–Cr_1_–N_2_	78.63(10)	Cr_1_–Cl_2_	2.3284(10)
O_2_–Cr_1_–N_1_	171.10(10)		
O_1_–Cr_1_–N_2_	174.03(10)		
