# Supplementary material for: Synthesis, Characterization, and Antifungal Studies of Cr(III) Complex of Norfloxacin and Bipiridyl Ligand
Source: Bioinorg Chem Appl. 2014 Sep 3;2014:457478. doi: 10.1155/2014/457478 (PMC4168151; doi:10.1155/2014/457478)
Supplement: Supplementary file 1 — S1: FT-IR spectra of (a) Nor and (b) [Cr(Nor)(Bipy)Cl2]Cl.2CH3OH S2: Electronic spectra of (a) Nor and (b) [Cr(Nor)(Bipy)Cl2]Cl.2CH3OH [file 457478.f1.zip › mat.457478.v2.pdf]

<u>**Supplementary material file**</u>

Synthesis, Characterization and Antifungal Studies of Cr(III) complex of Norfloxacin and Bipyridyl Ligand

Debnath Anamika[a], Hussain Firasat[a] and Masram Dhanraj T.[a]*

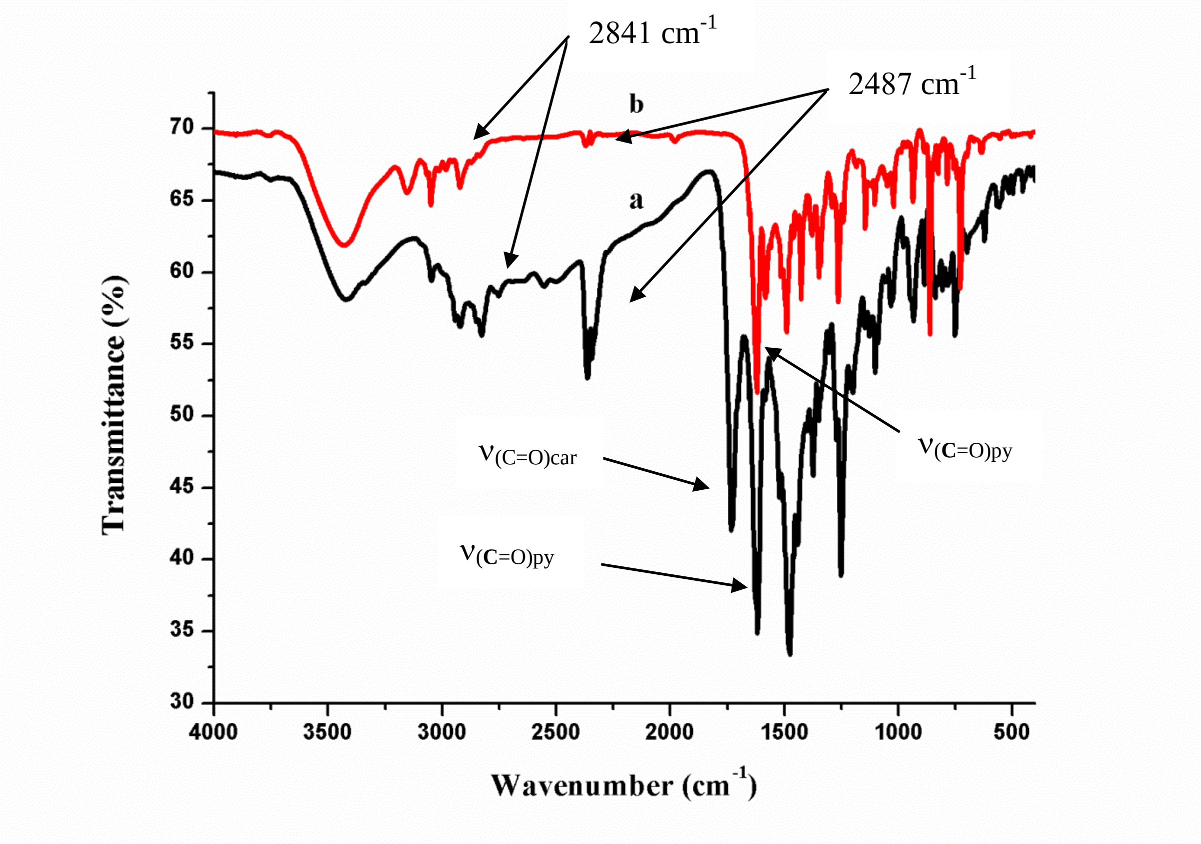

S1: FT-IR spectra of (a) Nor and (b) [Cr(Nor)(Bipy)Cl_2]Cl.2CH_3OH

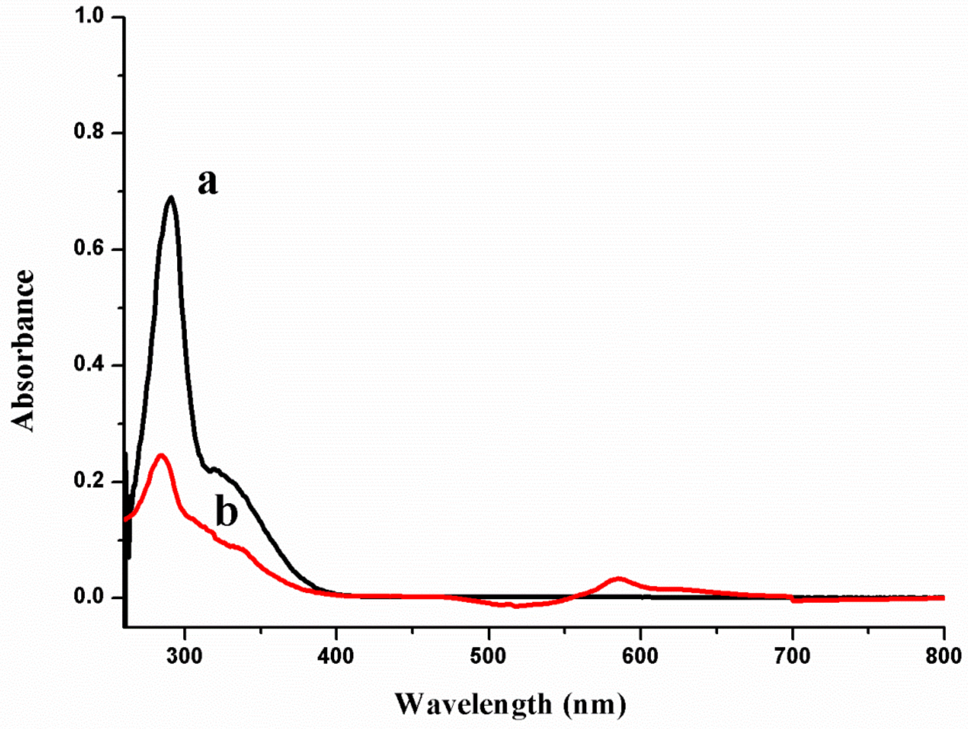

S2: Electronic spectra of (a) Nor and (b) [Cr(Nor)(Bipy)Cl_2]Cl.2CH_3OH
