# Supplementary material for: Synthesis, Characterization, and Antifungal Studies of Cr(III) Complex of Norfloxacin and Bipiridyl Ligand
Source: Bioinorg Chem Appl. 2014 Sep 3;2014:457478. doi: 10.1155/2014/457478 (PMC4168151; doi:10.1155/2014/457478)
Supplement: Supplementary file 1 — S1: FT-IR spectra of (a) Nor and (b) [Cr(Nor)(Bipy)Cl2]Cl.2CH3OH S2: Electronic spectra of (a) Nor and (b) [Cr(Nor)(Bipy)Cl2]Cl.2CH3OH [file 457478.f1.zip › suppl. description.docx]

## S1: FT-IR spectra of (a) Nor and (b) [Cr(Nor)(Bipy)Cl2]Cl.2CH3OH

## For FT-IR: Δ *ν* (C=O)carb, was replaced in the complex by two characteristic bands at 1587 cm−1 and at 1381 cm−1assigned as asymmetric *ν* (O–C–O)a and symmetric *ν* (O– C–O)s stretching vibrations, respectively. This indicates the involvement of the pyridone oxygen and carboxylate oxygen in the coordination with Cr(III) ion. The difference Δ *ν* = *ν* (O–C–O)a − *ν* (O–C–O)s is the important criteria for the determination of coordination mode of the ligand [32]. Δ *ν* of the complex was found to be 206 cm−1 that indicates the monodentate interaction of the carboxylate group with metal ion.

## S2: Electronic spectra of (a) Nor and (b) [Cr(Nor)(Bipy)Cl2]Cl.2CH3OH

## For Electronic spectra: Two bands have been found at 285 nm and 335 nm in case of free ligand. These two bands were assigned to 𝜋-𝜋∗ and 𝑛-𝜋∗ transitions, respectively. These two transitions were observed due to the presence of aromatic ring containing pyridone oxygen and carboxylate oxygen. Pattern of the electronic spectra of Cr(III) complex is similar to that of the free ligand, indicating that the ligand has not changed its structure during complexation. A broad band was observed in the visible region which is centred at 590 nmdue to d-d transition.
